# The addition of tislelizumab to gemcitabine and cisplatin chemotherapy increases thrombocytopenia in patients with urothelial carcinoma: A single‐center study based on propensity score matching

**DOI:** 10.1002/cam4.6807

**Published:** 2023-12-13

**Authors:** Zhimin Gao, Nienie Qi, Xu Qin, Zhen Li, Gang Li, Zewei Wang, Junqi Wang, Rumin Wen, Hailong Li

**Affiliations:** ^1^ Department of Urology The Affiliated Hospital of Xuzhou Medical University Xuzhou China; ^2^ Graduate School of Xuzhou Medical University Xuzhou China

**Keywords:** cisplatin, gemcitabine, myelosuppression, risk factors, tislelizumab, urothelial carcinoma

## Abstract

**Purpose:**

Whether the addition of tislelizumab to gemcitabine and cisplatin (GC) chemotherapy increases the incidence of myelosuppression has not been well established. This study identified the risk factors for the development of myelosuppression in patients with urothelial carcinoma (UC) after receiving GC chemotherapy with or without tislelizumab.

**Materials and Methods:**

We enrolled 192 UC patients who received GC with or without tislelizumab at the Affiliated Hospital of Xuzhou Medical University between July 2014 and November 2022. Patient baseline characteristics were included in the statistical analyses after adjusting for previously reported risk factors affecting survival using propensity score matching (1:1). Binary logistic regression analysis was used to identify the risk factors associated with posttreatment myelosuppression.

**Results:**

A total of 192 patients were enrolled, of whom 96 were treated with tislelizumab plus gemcitabine and cisplatin (T + GC) and 96 with GC alone. The incidence of leukopenia, anemia, and thrombocytopenia of any grade was 50.0%, 70.8%, and 42.7%, respectively, in the T + GC group and 41.7%, 72.9%, and 20.8%, respectively, in the GC group. In multivariate analysis, patients aged over 70 years (OR = 2.486, 95% CI: 1.067–5.792, *p* = 0.035) and those who received T + GC (OR = 3.119, 95% CI: 1.576–6.173, *p* = 0.001) were more likely to develop thrombocytopenia. Patients aged over 70 years (OR = 3.213, 95% CI: 1.254–8.237, *p* = 0.015) were more likely to develop anemia, and patients with renal insufficiency (OR = 2.105, 95% CI: 1.035–4.280, *p* = 0.040) were more likely to develop leukopenia. Eventually, 99 (51.6%) patients with UC successfully completed all the treatment cycles.

**Conclusions:**

This study demonstrates that the addition of tislelizumab to GC chemotherapy led to a considerable increase in the occurrence of thrombocytopenia, whereas no significant changes were observed regarding anemia or leukopenia. It is crucial to fully inform patients at increased risk for myelosuppression of potential risks and closely monitor changes in their blood routines.

## 1 INTRODUCTION

Platinum‐based chemotherapy has been the first‐line treatment for advanced urothelial carcinoma (UC) since 1980.^1^ Gemcitabine and cisplatin (GC) therapy is considered the standard treatment because of its similar survival benefit and better safety profile compared to methotrexate, vinblastine, doxorubicin, and cisplatin (MVAC).^2^, ^3^ The European Association of Urology (EAU) guidelines recommend neoadjuvant chemotherapy (NAC) for patients with T2‐T4aN0M0 bladder cancer (BC) or postoperative adjuvant chemotherapy (AC) if NAC is not administered.^4^ Neoadjuvant cisplatin‐based chemotherapy was associated with a statistically significant benefit (*p* = 0.004) regarding overall survival (OS).^5^ In addition, the EAU guidelines recommend postoperative AC for pT2‐4 or N+ upper tract urothelial carcinoma (UTUC),^6^ and a study based on a large sample size has shown a significant OS benefit for patients with UTUC receiving postoperative AC (*p* < 0.001).^7^ Despite the impressive efficacy of GC in the treatment of advanced UC, treatment‐related adverse events (TRAEs) should not be ignored. A phase III trial has revealed that the most common grade 3 or 4 toxicities were hematological toxicities, with approximately 55% of muscle‐invasive bladder cancer (MIBC) patients experiencing grade ≥3 hematological toxicities after GC therapy.^8^ Another phase III study has described that, in advanced or metastatic BC, grade 3 or higher neutropenia, thrombocytopenia, and anemia occurred in 71%, 57%, and 27% of patients, respectively, and 37% of GC cycles were administered with dose adjustments owing to hematological toxicities and renal insufficiency.^9^

Recently, with the advent of immune checkpoint inhibitors (ICIs), more options have become available for treating UC. With the approval of tislelizumab for the treatment of advanced UC in China, an increasing number of studies have focused on the efficacy of tislelizumab plus gemcitabine and cisplatin (T + GC). However, no studies have investigated whether the addition of tislelizumab to GC therapy increases the incidence of myelosuppression in patients with UC.

This study assessed whether T + GC therapy significantly increased the incidence of myelosuppression in patients with UC compared to those treated with GC alone and evaluated the risk factors for myelosuppression in patients with UC treated with GC or T + GC using propensity score matching (PSM).

## 2 MATERIALS AND METHODS

### 2.1 Patients Characteristics

We examined 305 patients with UC who were treated with T + GC or GC between July 2014 and November 2022 at our institution. GC chemotherapy was initiated in July 2014. The combination of tislelizumab and GC was administered in April 2020. Eligible patients had histologically or cytologically confirmed UC of the renal pelvis, ureter, or bladder^10^; had not received prior systemic immunotherapy or chemotherapy for locally advanced or metastatic UC; and had an Eastern Cooperative Oncology Group performance score (ECOG‐PS) of 0 or 1. Adequate hematological data were required, including baseline hematological data within 7 days before initiating T + GC or GC therapy and posttreatment routine blood tests.

Patients with active autoimmune disease and those who have received systemic treatment with immunomodulatory drugs or corticosteroids within 1 year before the start of treatment were excluded. According to the Galsky criteria,^11^ patients deemed ineligible for cisplatin‐based chemotherapy should also be excluded. This retrospective single‐institution study was approved by the Ethics Committee of the Affiliated Hospital of Xuzhou Medical University. Written informed consent was obtained from all patients.

### 2.2 Data collection

We recorded the following baseline characteristics of patients with UC by consulting their electronic medical records: age, sex, body mass index (BMI), smoking history, hypertension, diabetes, hydronephrosis, ECOG‐PS, primary tumor site (renal pelvis/ureter/bladder), renal function, and whether radical surgery was performed. Relevant laboratory data were collected, including white blood cell (WBC) count, hemoglobin, and platelet count, the lower normal limits of which are leucocytes 4 × 10^9^ cells per L, hemoglobin 12 g/dL; and platelets 100 × 10^9^ cells per L. The diagnostic criteria for renal insufficiency required both a serum creatinine level > 133 μmol/L and glomerular filtration rate (eGFR) >60 mL/min. We determined the grade of TRAEs using the Common Terminology Criteria for Adverse Events (CTCAE) Version 4.03 of the National Cancer Institute.

### 2.3 Treatment and Evaluations

Patients received 21‐day cycles of gemcitabine (1000 mg/m^2^ body surface area, administered intravenously on days 1 and 8 of each cycle) and cisplatin (70 mg/m^2^ body surface area administered intravenously on day 1 of each cycle) with or without tislelizumab (200 mg administered intravenously on day 1 of each cycle) as first‐line therapy. The specific treatment regimen for T + GC or GC was determined by the physician and patient based on the circumstances of the individual patient, and the treatment cycles varied according to the different stages of UC. For locally advanced UC, postoperative adjuvant therapy was administered for four cycles, whereas six cycles of systemic therapy were recommended for metastatic UC. Patients with UC were treated with T + GC or GC until completion of all cycles of treatment, disease progression, intolerable toxicity effects, or withdrawal of consent.

### 2.4 Statistical analyses

All variables were transformed into categorical variables based on routine cutoff points in clinical applications. Categorical variables were expressed as counts with percentages and compared using the *χ* ^2^ test. In univariate logistic regression analysis, we included variables with *p* < 0.2, as well as the concerned “treatment regimen” in this study, into the subsequent multivariate logistic regression. The results were reported as odds ratios (OR) with 95% confidence intervals (CI).

To reduce confounding factors, a propensity score was used to match patients treated with GC to those treated with T + GC. The following variables were matched by calculating the propensity score for each patient, such as age, sex, BMI, smoking history, hypertension, diabetes, hydronephrosis, ECOG‐PS, primary site, renal function, and surgical removal of the primary tumor. PSM was performed using the one‐to‐one matching method, and the caliper was determined before analysis so that the standardized difference in all confounding factors was <0.25.^12^ Differences before and after matching were assessed using the *χ* ^2^ test.

All statistical analyses were two‐sided, and statistical significance was set at *p* < 0.05. Statistical analysis was performed using SPSS (version 26.0; IBM Corp.) and PSM using R version 4.0.5 (R Foundation for Statistical Computing).

## 3 RESULTS

A total of 305 patients were enrolled at our institution between July 2014 and November 2022, including 190 (62.3%) in the GC group and 115 (37.7%) in the T + GC group. Before 1:1 PSM, statistically significant differences were observed in sex, hydronephrosis, tumor site, and surgical removal of the primary tumor. Compared to the T + GC group, a minority of patients in the GC group developed hydronephrosis (T + GC 60.9%; GC 39.5%). At the time of data cutoff, 99 (52.1%) patients completed all cycles of GC, and 62 (53.9%) patients completed all cycles of T + GC.

After PSM, 192 patients treated with T + GC or GC were matched, with non‐significant differences in all confounding factors between the two groups (Table 1). In both groups, the majority of patients were males (T + GC 69.8%; GC 70.8%) and current or former smokers (T + GC 66.7%; GC 69.8%). Further, 83 (86.5%) patients were treated with adjuvant GC chemotherapy after radical resection, and 84 (87.5%) patients were treated with T + GC postoperatively. The percentages of patients with UTUC and BC were 64.6% and 35.4%, respectively, in the GC group and 51.0% and 49.0%, respectively, in the T + GC group. Additionally, 48 (50.0%) patients completed all cycles of GC, and 51 (53.1%) completed all cycles of T + GC.

**TABLE 1:** Patient demographics and baseline disease characteristics (GC or T + GC).

Variables	Before PSM		After PSM		*p* Value	
	GC (*n* = 190)	T + GC (*n* = 115)	GC (*n* = 96)	T + GC (*N* = 96)	Pre‐PSM	Post‐PSM
Median age (range), years	62 (29–84)	63 (34–84)	64 (32–84)	63 (34–84)		
Age group, *n* (%)					0.060	0.537
≤70	137 (72.1)	71 (61.7)	67 (69.8)	63 (65.6)		
>70	53 (27.9)	44 (38.3)	29 (30.2)	33 (34.4)		
Sex, *n* (%)					0.014	0.874
Female	36 (18.9)	36 (31.3)	28 (29.2)	29 (30.2)		
Male	154 (81.1)	79 (68.7)	68 (70.8)	67 (69.8)		
BMI, *n* (%)					0.925	0.562
<25	108 (56.8)	66 (57.4)	51 (53.1)	55 (57.3)		
≥25	82 (43.2)	49 (42.6)	45 (46.9)	41 (42.7)		
Smoking history, *n* (%)					0.534	0.642
No	66 (34.7)	44 (38.3)	29 (30.2)	32 (33.3)		
Yes	124 (65.3)	71 (61.7)	67 (69.8)	64 (66.7)		
Hypertension, *n* (%)					0.128	0.735
No	153 (80.5)	84 (73.0)	72 (75.0)	74 (77.1)		
Yes	37 (19.5)	31 (27.0)	24 (25.0)	22 (22.9)		
Diabetes, *n* (%)					0.069	1.000
No	177 (93.2)	100 (87.0)	88 (91.7)	88 (91.7)		
Yes	13 (6.8)	15 (13.0)	8 (8.3)	8 (8.3)		
Hydronephrosis, *n* (%)					<0.001	0.884
No	115 (60.5)	45 (39.1)	40 (41.7)	41 (42.7)		
Yes	75 (39.5)	70 (60.9)	56 (58.3)	55 (57.3)		
ECOG status, *n* (%)					0.392	0.739
0	130 (68.4)	84 (73.0)	73 (76.0)	71 (74.0)		
1	60 (31.6)	31 (27.0)	23 (24.0)	25 (26.0)		
Primary tumor site, *n* (%)					0.001	0.057
UTUC	62 (32.6)	59 (51.3)	62 (64.6)	49 (51.0)		
BC	128 (67.4)	56 (48.7)	34 (35.4)	47 (49.0)		
Surgical removal of primary site, *n* (%)					0.011	0.830
No	91 (47.9)	38 (33.1)	13 (13.5)	20 (20.8)		
Yes	99 (52.1)	77 (66.9)	83 (86.5)	76 (79.2)		
Renal dysfunction, *n* (%)					0.090	0.324
No	163 (85.8)	90 (78.3)	69 (71.9)	74 (77.1)		
Yes	27 (14.2)	25 (21.7)	27 (28.1)	22 (22.9)		

Abbreviations: PSM, propensity score matching; GC, gemcitabine and cisplatin; T+GC, tislelizumab plus gemcitabine and cisplatin; BMI, body mass index; ECOG, Eastern Cooperative Oncology Group; UTUC, upper tract urothelial carcinoma; BC, bladder cancer.

Of the 192 patients treated with T + GC or GC, 88 (45.8%) developed leukopenia during treatment. The incidence of leukopenia was 41.7% and 50.0% in the GC and T + GC groups, respectively (Table 2). After only one cycle of treatment, leukopenia occurred in 15 (15.6%) patients treated with GC and 21 (21.9%) patients treated with T + GC. Four (2.1%) patients (two in the T + GC group and two in the GC group) discontinued treatment permanently because of severe leukopenia, and eight (4.2%) patients underwent dose adjustment. Multivariate logistic regression analysis showed that leukopenia was associated with renal insufficiency (OR = 2.105, 95% CI: 1.035–4.280, *p* = 0.040) before treatment (Table 3). Addition of tislelizumab to GC chemotherapy did not increase the incidence of leukopenia in patients with UC.

**TABLE 2:** Hematological toxicities reported for patients in the GC and T + GC arms.

	GC (*n* = 96)	T + GC (*n* = 96)	*p*‐Value
Anemia	70 (72.9%)	68 (70.8%)	0.748
Leukopenia	40 (41.7%)	48 (50.0%)	0.247
Thrombocytopenia	20 (20.8%)	41 (42.7%)	0.001

*Note*: Data are presented as frequency (percentage). Comparisons between the GC and T + GC groups are performed with a chi‐square test. A *p*‐value < 0.05 would assume a statistical difference between the GC and T + GC groups.

Abbreviations: GC, gemcitabine and cisplatin; T + GC, tislelizumab plus gemcitabine and cisplatin chemotherapy.

**TABLE 3:** Logistic regression model of factors predicting leukopenia.

	Univariate analysis		Multivariate analysis	
Variables	OR (95% CI)	*p*	OR (95% CI)	*p*
Age				
≤70	1			
>70	1.058 (0.577–1.940)	0.857		
BMI				
<25	1			
≥25	0.748 (0.421–1.326)	0.320		
Smoking history				
Never	1			
Ever	0.821 (0.447–1.509)	0.526		
Hydronephrosis				
No	1			
Yes	1.011 (0.569–1.796)	0.971		
ECOG status				
0	1			
1	1.000 (0.519–1.926)	1.000		
Primary tumor site				
UTUC	1			
BC	1.396 (0.785–2.482)	0.256		
Surgical removal of primary site				
No	1			
Yes	1.596 (0.668–3.814)	0.293		
Renal dysfunction				
No	1		1	
Yes	1.739 (0.908–3.332)	0.095	2.105 (1.035–4.280)	0.040
Treatment regimen				
GC	1			
T + GC	1.400 (0.792–2.475)	0.247		
Chemotherapy cycle				
1	1			
≥2	0.940 (0.527–1.677)	0.834		

Most patients (*n* = 138; 71.9%) in both groups developed anemia during the treatment period. The prevalence of anemia was 72.9% and 70.8% in the GC and T + GC groups, respectively (Table 2). A total of 43.8% (T + GC, *n* = 42; GC, *n* = 42) of patients developed anemia after one cycle of treatment. Most patients with mild anemia return to normal and continue the next cycle of treatment. Additionally, 18 (9.4%) patients were treated with recombinant human erythropoietin or received dose adjustments for moderate anemia. None of the patients received red blood cell transfusions or permanently discontinued treatment because of severe anemia. Only 26.5% (T + GC, *n* = 26; GC, *n* = 25) of the patients remained free of anemia after receiving all cycles of treatment, and the remaining three (1.6%) patients without anemia terminated treatment because of other TRAEs. Multivariate logistic regression analysis showed that anemia was associated with age (OR = 3.213, 95% CI: 1.254–8.237, *p* = 0.015) in patients with UC (Table 4). Compared with GC, T + GC did not significantly increase the incidence of anemia in patients with UC.

**TABLE 4:** Logistic regression model of factors predicting anemia.

	Univariate analysis		Multivariate analysis	
Variables	OR (95% CI)	*p*	OR (95% CI)	*p*
Age				
≤70	1		1	
>70	2.660 (1.234–5.735)	0.013	3.213 (1.254–8.237)	0.015
BMI				
<25	1			
≥25	1.399 (0.737–2.657)	0.304		
Smoking history				
Never	1			
Ever	1.241 (0.638–2.415)	0.525		
Hydronephrosis				
No	1			
Yes	0.921 (0.486–1.744)	0.800		
ECOG status				
0	1			
1	1.672 (0.765–3.653)	0.197		
Primary tumor site				
UTUC	1			
BC	0.977 (0.517–1.847)	0.943		
Surgical removal of primary site				
No	1			
Yes	1.238 (0.500–3.064)	0.644		
Renal dysfunction				
No	1			
Yes	1.540 (0.721–3.289)	0.265		
Treatment regimen				
GC	1			
T + GC	0.902 (0.481–1.693)	0.748		
Chemotherapy cycle				
1	1			
≥2	1.956 (1.034–3.701)	0.039		

In both groups, posttreatment thrombocytopenia occurred in a minority of patients (*n* = 61; 31.8%) with UC. During follow‐up, 20 (20.8%) and 41 (42.7%) patients developed thrombocytopenia after GC and T + GC therapy, respectively (Table 2). Compared to the GC group, more patients (T + GC, *n* = 21; GC, *n* = 10) developed thrombocytopenia after only one cycle of T + GC. Regardless of whether oral or injectable drugs were used to stimulate thrombocytopoiesis, most patients (*n* = 59; 96.7%) were able to tolerate multiple cycles of T + GC or GC in patients who developed thrombocytopenia. However, three (3.1%) patients in the GC group underwent dose adjustment because of severe thrombocytopenia, and T + GC therapy was permanently terminated in two (2.1%) patients. Multivariate logistic regression analysis showed that thrombocytopenia was associated with age (OR = 2.486, 95% CI: 1.067–5.792, *p* = 0.035) and addition of tislelizumab (OR = 3.119, 95% CI: 1.576–6.173, *p* = 0.001) to GC therapy (Table 5). For patients with UC, those aged >70 years and those who received T + GC were more likely to develop thrombocytopenia.

**TABLE 5:** Logistic regression model of factors predicting thrombocytopenia.

	Univariate analysis		Multivariate analysis	
Variables	OR (95%CI)	*p*	OR (95%CI)	*p*
Age				
≤70	1		1	
>70	1.764 (0.934–3.334)	0.080	2.486 (1.067–5.792)	0.035
BMI				
<25	1			
≥25	1.177 (0.639–2.165)	0.601		
Smoking history				
Never	1			
Ever	1.660 (0.837–3.290)	0.147		
Hydronephrosis				
No	1			
Yes	0.883 (0.478–1.631)	0.691		
ECOG status				
0	1			
1	0.850 (0.416–1.734)	0.655		
Primary tumor site				
UTUC	1			
BC	0.762 (0.409–1.419)	0.391		
Surgical removal of primary site				
No	1			
Yes	1.229 (0.484–3.119)	0.665		
Renal dysfunction				
No	1			
Yes	1.147 (0.579–2.276)	0.694		
Treatment regimen				
GC	1		1	
T + GC	2.833 (1.498–5.358)	0.001	3.119 (1.576–6.173)	0.001
Chemotherapy cycle				
1	1			
≥2	1.027 (0.528–1.906)	0.064		

## 4 DISCUSSION

As research progresses, more treatment options have become available for patients with advanced UC. In the last decade, ICIs have revolutionized the approach to cancer treatment. The National Comprehensive Cancer Network (NCCN) guidelines recommend ICIs, such as pembrolizumab and atezolizumab, for the first‐ or second‐line treatment of locally advanced or metastatic UC.^13^ Previous studies have indicated that ICI in combination with chemotherapy as a first‐line treatment may provide favorable treatment outcomes for patients with UC.^14^, ^15^ In the IMvigor130 trial,^14^ adding atezolizumab to platinum‐based chemotherapy was found to prolong progression‐free survival (PFS) in the treatment of metastatic UC (*p* = 0.007), whereas there was no significant improvement in the median OS.

Tislelizumab is a humanized immunoglobulin G4 monoclonal antibody designed to bind to the programmed cell death‐1 (PD‐1) receptor with higher affinity than other anti‐PD‐1 drugs.^16^ In addition, the structure of tislelizumab has been specifically modified to minimize the binding of tislelizumab to Fcγ receptors on macrophages.^17^ Attenuation of this binding can maximally inhibit antibody‐dependent phagocytosis, which is a potential mechanism for T‐cell clearance and resistance to anti‐PD‐1 therapy.^18^ In April 2020, the Chinese National Medical Products Administration approved tislelizumab for the treatment of advanced UC with high programmed cell death protein ligand 1 (PD‐L1) expression, opening a new era of immunotherapy for UC in China. Therefore, the incidence of TRAEs associated with tislelizumab monotherapy should not be overlooked. The most commonly reported TRAEs induced by tislelizumab monotherapy were grade 1–2 in severity, with anemia (27%) and pyrexia (19%) being the most common.^19^ Immune‐related adverse reactions (irAEs) occurred in 27% of patients with UC, and the common irAEs were skin reactions (12%), hypothyroidism (11%), and hyperthyroidism (6%).

A phase II clinical study in China showed that tislelizumab monotherapy had an objective response rate (ORR) of 24% and a disease control rate (DCR) of 38% in patients with PD‐L1‐positive advanced or metastatic UC.^19^ However, conventional platinum‐based chemotherapy regimens show a response rate of at least 45% in patients with metastatic UC.^9^ Therefore, for patients with UC, tislelizumab monotherapy is not a replacement for first‐line chemotherapy but provides a promising treatment option for patients who cannot tolerate cisplatin‐based chemotherapy. In this multicenter retrospective study,^20^ 253 patients with MIBC were enrolled to compare the efficacies of neoadjuvant immunotherapy (tislelizumab), NAC (GC), and neoadjuvant combination therapy (T + GC). The results showed that, compared with the NAC and neoadjuvant immunotherapy cohorts, patients treated with neoadjuvant T + GC achieved a significantly higher complete response rate (*p* = 0.001) and pathological downstaging rate (*p* = 0.007). In addition, a series of TRUCE studies were designed to evaluate the efficacy and safety of neoadjuvant tislelizumab in combination with nab‐paclitaxel in both MIBC^21^ and high‐risk non‐muscle‐invasive urothelial bladder carcinoma (NMIBC).^22^ Researchers have found that the combination of tislelizumab and nab‐paclitaxel provides a novel neoadjuvant therapeutic option with a satisfactory efficacy and safety profile.^21^ Another retrospective study was performed to assess the efficacy of T + GC as a first‐line adjuvant treatment in patients with locally advanced or metastatic BC.^23^ The results showed that the median PFS in the T + GC group was longer than that in the GC group (*p* = 0.004). A possible reason for this may be the chemotherapy‐mediated alteration of the tumor microenvironment, which increases tumor cell immunogenicity and T‐lymphocyte infiltration, thereby enhancing the therapeutic effect of ICIs.^24^ A phase III randomized controlled trial, BGB‐A317‐310, of first‐line tislelizumab combined with platinum‐based chemotherapy for the treatment of locally advanced or metastatic UC in the Chinese population is ongoing.

The previous studies have focused on the efficacy of tislelizumab in combination with chemotherapy. However, no studies have been conducted to determine whether adding tislelizumab to GC chemotherapy increases the incidence of myelosuppression. In this study, the incidence of leukopenia, anemia, and thrombocytopenia of any grade was 50.0%, 70.8%, and 42.7%, respectively, in the T + GC group and 41.7%, 72.9%, and 20.8%, respectively, in the GC group. We found that patients aged >70 years were more likely to develop anemia and thrombocytopenia. Although stem cell reserves are not depleted with age, the reserves and functions of stem cells continue to decline.^25^ Regarding GC chemotherapy or T + GC immunochemotherapy, the functional capacity of stem cells is severely impaired, resulting in the loss of proliferation potential and self‐renewal ability of hemoglobin and platelets.^26^ In addition, we found that patients treated with T + GC were more likely to develop thrombocytopenia. That is, the addition of tislelizumab to conventional GC regimen did not significantly increase the risk of anemia and leukopenia but significantly increased the incidence of thrombocytopenia. For patients receiving ICI plus platinum‐based chemotherapy, myelosuppression is the predominant TRAE, which is largely attributed to the cytotoxicity of platinum‐based anticancer drugs.^27^ Regarding myelosuppression, thrombocytopenia of all grades was more likely to occur in patients treated with T + GC. Although we are currently unable to clearly explain the possible reasons for this correlation, early onset of myelosuppression is insidious. Therefore, our study is critical to guiding clinical practice. By identifying the risk factors for the development of myelosuppression, clinicians should pay more attention to and review routine posttreatment blood tests more frequently for patients with high‐risk myelosuppression. Based on the findings of this study, clinicians may proactively prevent or relieve severe myelosuppression by prophylactically administering cell proliferation‐stimulating agents or promptly adjusting the medication dosage.

This study had some limitations. First, it was retrospective, and an insufficient sample size may have influenced the assessment of the correlation between baseline patient characteristics and myelosuppression. Second, we recruited individuals who received medical interventions between 2014 and 2022. As medical treatments are subject to alterations in options, cycles, and strategies over time, such modifications may affect outcomes. Third, although PSM was used to adjust for risk factors, we cannot rule out other unknown confounding factors that may have contributed to the bias. Last, we did not collect data on patients treated with tislelizumab monotherapy at our institution; therefore, we could not directly compare the incidence of myelosuppression after receiving single‐agent tislelizumab and T + GC. Further research with a larger population is required to strengthen and validate our findings.

## 5 CONCLUSIONS

This study demonstrated that the addition of tislelizumab to GC chemotherapy led to a considerable increase in the incidence of thrombocytopenia, whereas no significant alterations were observed regarding anemia or leukopenia. For individuals at an elevated risk of myelosuppression, it is imperative to fully inform them of the potential risks and closely monitor changes in their blood routines.

## AUTHOR CONTRIBUTIONS

**Zhimin Gao:** Writing – original draft (lead). **Nienie Qi:** Writing – review and editing (equal). **Xu Qin:** Data curation (equal). **Zhen Li:** Resources (equal). **Gang Li:** Data curation (equal). **Zewei Wang:** Data curation (equal). **Junqi Wang:** Project administration (equal). **Rumin Wen:** Supervision (equal). **Hailong Li:** Funding acquisition (equal); writing – review and editing (equal).

## FUNDING INFORMATION

This study was sponsored by Six talent peaks project in Jiangsu Province [grant number WSW‐064]; Project supported by the Natural Science Foundation of the Jiangsu Higher Education Institutions of China [grant number 19KJB310001]; Post‐doctoral Research Funding Project in Jiangsu Province [grant number 2021K447C] and Second Round of Xuzhou Medical Leading Talents Training Project [grant number XWRCHT20210027]; The Science and Technology Planning Project of Traditional Chinese Medicine of Jiangsu [grant number YB2020050]; Practice Innovation Program of Jiangsu Province [grant number KYCX21_2641]; Postgraduate Research and Practice Innovation Project of Jiangsu Province [grant number KYCX23‐2926];

## CONFLICT OF INTEREST STATEMENT

The authors declare no competing interests.

## ETHICS STATEMENT

This retrospective single‐institutional study was approved by the ethics committee of the Affiliated Hospital of Xuzhou Medical University (XYFY2022‐KL340). All patients provided written informed consent.

## CONSENT FOR PUBLICATION

Informed consent to publish was obtained.

## Data Availability

The dataset analyzed in this study is available from the corresponding author upon reasonable request.
